# Long non-coding RNA LINC00616 promotes ferroptosis of periodontal ligament stem cells via the microRNA-370 / transferrin receptor axis

**DOI:** 10.1080/21655979.2022.2076508

**Published:** 2022-05-25

**Authors:** Hongwei Wang, Xiaotong Qiao, Chao Zhang, Jingyi Hou, Suqing Qi

**Affiliations:** a Department of Orthodontics, Eye Hospital of Hebei, Xingtai, Hebei, China; b Department of Oral Medicine, College of Stomatology, Hebei Medical University, Shijiazhuang, Hebei, China; c Department of Orthodontics, Beijing Stomatological Hospital, Capital Medical University, Beijing, China

**Keywords:** Periodontitis, bone marrow mesenchymal stem cells, LINC00616, ferroptosis, miR-370, *TFRC*

## Abstract

This study was designed to explore the role of lncRNA LINC00616 in the regulation of periodontitis. Cellular functions were measured using 3-(4,5-dimethylthiazol-2-yl)-2,5-diphenyltetrazolium bromide (MTT) and terminal deoxynucleotidyl transferase-mediated dUTP nick end labeling (TUNEL) assays. The content of reactive oxygen species, Fe^2+^, glutathione, and malondialdehyde were measured to determine ferroptosis in *Porphyromonas gingivalis* lipopolysaccharide (LPS-PG) treated periodontal ligament stem cells (PDLSCs), as well as expression of glutathione peroxidase 4 (GPX4), solute carrier family 7 member 11, and acyl-CoA synthetase long-chain family member 4 proteins mRNA and miRNA levels were measured by quantitative reverse-transcription polymerase chain reaction (qRT-PCR). Western blot analysis was performed to assess protein expression. Targeting relationships were predicted using StarBase and TargetScan and verified by a dual luciferase reporter assay. The lncRNA LINC00616 was upregulated in periodontitis ligament tissues of patients with periodontitis and in PDLSCs treated with LPS-PG. Inhibition of LINC00616 promoted cell viability and suppressed ferroptosis of PDLSCs. miR-370 was verified to be a target of LINC00616, and suppressed miR-370 reversed the effects of LINC00616 knockdown on cell viability and ferroptosis in PDLSCs. Additionally, miR-370 targeting the transferrin receptor protein and upregulated transferrin receptor (TFRC) abolished the effects of overexpressed miR-370 on cell viability and ferroptosis of PDLSCs. LINC00616 acted as a competitive endogenous RNA (ceRNA) to promote ferroptosis of PDLSCs via the miR-370/TFRC axis. Therefore, LINC00616 knockdown may be a promising therapeutic strategy for periodontitis.

## graphical abstract

**Figure:**
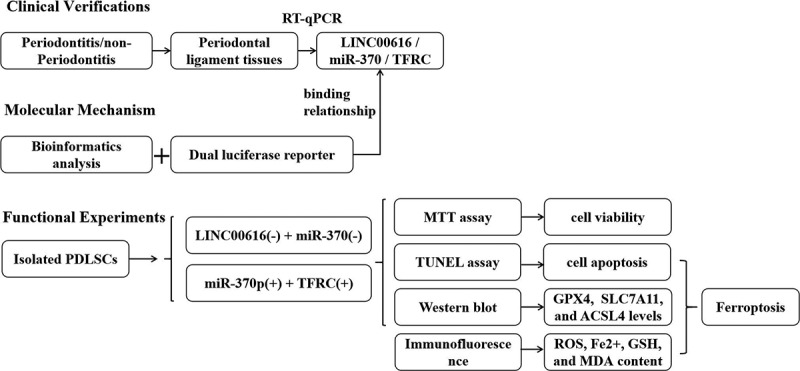

## Highlights

- lncRNA LINC00616 was upregulated in periodontitis.
- Inhibition of LINC00616 promoted cell viability and suppressed ferroptosis of PDLSCs.
- LINC00616 acted as a ceRNA to promote ferroptosis of PDLSCs via miR-370/TFRC axis.
- LINC00616 knockdown may be a promising therapeutic strategy for periodontitis.

## Introduction

Chronic periodontitis is a common chronic inflammatory disease with oral pathogenic microorganisms as an initiating factor and the formation of the periodontal pocket and resorption of the alveolar bone as the main clinical features [1,2]. Current sequential periodontal therapy and guided tissue regeneration in the treatment of periodontitis have corresponding limitations and cannot achieve physiological and functional regeneration of periodontal tissue [3,4].

Ferroptosis is a regulated cell death process caused by a redox state disorder of the intracellular microenvironment controlled by glutathione peroxidase 4 (GPX4) due to intracellular iron overload [5], which is usually accompanied by an increase in reactive oxygen species (ROS) secretion, iron ions (Fe^2+^ and Fe^3+^), and malondialdehyde (MDA), and a decrease in glutathione (GSH) secretion [6–8]. As we all know, ferroptosis regulates tumors and brain diseases [9–11]. Recently, researchers found that ferroptosis plays a regulatory role in inflammatory diseases, including periodontitis [12,13]. For example, lipopolysaccharide from P. gingivalis (LPS-PG), one major putative pathogen for periodontitis, may elicit mitochondrial dysfunction, high ROS production and oxidative stress [14]. Ferroptosis in periodontal ligament fibroblasts leads to development of periodontitis [13].

Long noncoding RNAs (lncRNAs) play a vital role in the initiation and progression of chronic periodontitis [15]. For example, microarray analysis revealed that 8925 lncRNAs were differentially expressed in periodontitis tissues compared with normal periodontal tissue, indicating that lncRNAs may play key roles in the course of periodontitis [16]. Furthermore, knockdown of lncRNA MEG3 inhibited the osteogenic differentiation of periodontal ligament stem cells (PDLSCs) in periodontitis [17]. Upregulation of lncRNA-01126 promoted the progress of periodontitis through the MEK/ERK signaling pathway [18]. Meanwhile, as one of the largest gene families, miRNAs are ncRNAs with a length of about 19–24 nucleic acids, which are widely found in eukaryotic cells [19]. The potential of miRNAs in biological processes and posttranscription confer regulatory function in chronic periodontitis [20]. Furthermore, lncRNAs function as a new type of competitive endogenous RNA (ceRNA) regulator through sponging miRNAs [21,22]. LINC00616 is a novel lncRNA identified by microarray analyses between periimplantitis and patients with periodontitis [23].

However, the underlying molecular mechanisms of LINC00616 in PDLSCs are yet to be investigated further. Our study aimed to investigate the role of LINC00616 in the development of periodontitis.

## Materials and methods

### Patients

Forty patients diagnosed with periodontitis at the Eye Hospital of Hebei and 40 non-periodontitis participants were included in this study with the approval of the Ethics Committee of Eye Hospital of Hebei (No. 2020KY019). This study was carried out in accordance with the Declaration of Helsinki. The inclusion criteria are as follows: 1) Patients with periodontitis need to have teeth extracted or patients without periodontitis need to have teeth extracted by orthodontic surgery; 2) All subjects have signed their informed consent after being fully informed; 3) All participants were over 18 years old. 4. The participants were free of any known systemic diseases.

### Periodontal ligament tissue

The tissue around the tooth was disinfected with 75% ethanol (Solarbio, China) and the samples were taken within 2 h after tooth extraction [24]. The root was rinsed 3–5 times with phosphate buffer solution (PBS; Solarbio, China) on a super clean table, and the periodontal ligament tissues were scraped in 1/3 of the root.

### Cell culture and establishment of periodontitis cell model

The tissues of the periodontal ligaments were minced into small pieces and treated with collagenase type I digestion (1 mg/mL; Sigma-Aldrich, NJ, USA) at 37°C for 0.25 h. The cell suspensions were then filtered and cultured in DMEM (Gibco, California, USA) containing 10% FBS (Gibco, California, USA), 2 mM L-glutamine (Gibco, California, USA), and 1% penicillin/streptomycin (Gibco, California, USA) [24]. Cells were digested after 14 days of incubation and single-cell-derived colony cultures were obtained using the limiting dilution technique. PDLSCs were obtained by dilution and passed five times.

The PDLSCs were divided into two groups, the control and LPS-PG groups. The final concentration of 1 μg/mL LPS-PG was added to the LPS-PG group [25–27]. Follow-up experiments were conducted after LPS-PG treatment 24 h later.

### Cell transfection

si-LINC00616 (si-LINC00616 1#, si-LINC00616 2#), miR-370 mimic, miR-370 inhibitor, overexpressed transferrin receptor protein plasmids (oe-TFRC), and their negative controls (nc) were purchased from GenePharma (Shanghai, China). The PDLSCs (2 × 10^5^ cells/well) were cultured in six-well plates. Cells were transfected into PDLSCs using Lipofectamine 3000 reagent (Life Technologies, California, USA) following the manufacturer’s protocol [28].

### Quantitative reverse transcription polymerase chain reaction (qRT-PCR)

qRT-PCR was used to detect miRNA and mRNA expression. Firstly, all RNA from tissues as well as PDLSCs were extracted using TRIzol reagent (Thermo Fisher Scientific, USA) and then dissolved in a solution without RNA enzyme. An ultraviolet spectrophotometer (NanoDrop, USA) was used to determine the ratio of A_260_/A_280_ RNA between 1.8 and 2.0. Next, 1 μg of qualified RNA was used to synthesize cDNA by reverse transcription (SuperScript First-Strand Synthesis Kit; Invitrogen, USA). The SYBR real-time fluorescence quantitative PCR kit (Takara, Japan) was used for fluorescence quantitative PCR analysis, and Primer BLAST (https://blast.ncbi.nlm.nih.gov/Blast.cgi) was used for primer design [29]. Reaction conditions: 94°C, 4 min 94°C, 30s 56°C, 30s 72°C, 30s, 40 cycles. GAPDH was used as a housekeeping control for LINC00616 and mRNAs. U6 was used as an internal reference for miR-370. Data were analyzed using the 2^−ΔΔCt^ relative expression method. All primer sequences were listed in Table 1. All experiments were repeated three times.

**Table 1.** Primer sequences

Gene	Primer
DPP10-A1	5’-CAGCCCTGATAGGCAGTAGAC-3’
	5’-TCATTGCCTTTGATCCTGCTGT-3’
RP11-1C8.7	5’-TGTTTTTCCCCAAGTGGCAC-3’
	5’-AGAGACATATCAGATTCCCCAATT-3’
GSE61474_XLOC_022835	5’-ACTTTTATGTGAAGCCCAGAGT-3’
	5’-TCCTGGTTGTTGCGGCTT-3’
RP11-142A22.4	5’-CTCTCCTGATGGTTGCACGA-3’
	5’-TGACAAAGCCAAGGCCAGAA-3’
G009665	5’-AGAACACCCCGATGACGGA-3’
	5’-GGCATCATTATGTACCCGGAAT-3’
MAFA-AS1	5’-GTGAATGAGGGCACAGCTCT-3’
	5’-CCCCAACTCCTCCCACTTTC-3’
LINC00616	5’-TGCAGCAGGCCACTATTTGA-3’
	5’-AGCCACTGAGATCAACTGCC-3’
G083982	5’-AGGTGAAGAGCACTACGAAGA-3’
	5’-CTACTATGACGCCACATGCCT-3’
RP4-660H19.1	5’-TAGGACCCTCCAAGCCAGG-3’
	5’-TTTCACAGGGAATGGTGGCC-3’
TRHDE-AS1	5’-CCTGAGAGCTTACTGCCACA-3’
	5’-CAACGCGCTCATCGAGAATG-3’
miR-370	5’-CTCACGGCCTGCTGGGGTG-3’
	5’-ACCTCAAGAACAGTATTTCCAGG-3’
TFRC	5’-TCGTGAGGCTGGATCTCAAAA-3’
	5’-CAATAGCCCAAGTAGCCAATCAT-3’
GAPDH	5’-AGCCACATCGCTCAGACAC-3’
	5’-GCCCAATACGACCAAATCC-3’
U6	5’-CTCGCTTCGGCAGCACA-3’
	5’-AACGCTTCACGAATTTGCGT-3’

### 3-(4,5-dimethylthiazol-2-yl)-2,5-diphenyltetrazolium bromide (MTT) assay

Cell viability was measured by the MTT assay. The PDLSCs were collected and 100 μL cell suspension (2 × 10^5^ cells/mL) was inoculated into 96-well plates; 20 μL MTT solution (5 mg/mL; Thermo Fisher Scientific, USA) was added to each well and cultured for 4 h (37°C, 5% CO_2)_. The culture medium was then discarded, 150 μL DMSO was added to each well and vibrated at low speed for 10 min [30]. Finally, the OD490 nm values of each group were detected by a microplate analyzer (NanoDrop, USA). The experiment was repeated three times.

### Determination of the content of ROS, Fe^2+^, GSH, and MDA

Levels of intracellular ROS, Fe^2+^, GSH, and MDA were measured separately using an oxidation-sensitive fluorescent probe DCFH-DA (D6883; Sigma, USA) [31], iron assay kit (ab83366, Abcam, USA), glutathione assay kit (CS0260; Sigma, USA), and lipid peroxidation assay kit (ab118970, Abcam, USA), respectively, according to the manufacturer’s instructions.

### Terminal deoxynucleotidyl transferase-mediated dUTP nick end labeling (TUNEL) assay

The death of PDLSC was detected using a TUNEL detection kit (ab66110, Abcam, USA) according to the manufacturer’s instructions [32]. PDLSCs were observed under a microscope (Nikon, Japan). Five fields were randomly selected under a 200-fold light microscope and the number of positive cells and total cells were counted.

### Western blot assay

The protein expressions of GPX4, solute carrier family 7 member 11 (SLC7A11), and acyl-CoA synthetase long-chain family member 4 (ACSL4) were detected by western blotting assay. Total protein samples were collected from the PDLSCs of each group and the protein concentration was determined by the BCA method (Sigma, USA). Proteins were resolved on 8% or 15% SDS-PAGE gels (100 V, 1.5–2 h) for separation and transferred to a polyvinylidene fluoride membrane. Primary antibodies (Abcam, USA) were added and incubated successively (overnight at 4°C, 12–16 h), and HRP labeled secondary antibody (at room temperature for 1 h) was added to avoid light for color development (31,460, Invitrogen, USA). The images were collected by an automatic gel imaging analyzer (BioRad, USA). All results were collected using Alpha image analysis software (Alpha Innotech, USA), and the absorbance value (A value) of the target band was analyzed and compared with the gray area value of GAPDH (ab8245, 1: 1000). The ratio of the two represents the relative expression level of the protein in the cell. The primary antibodies were as follows: anti-GPX4 (ab125066, 1: 1000), anti-SLC7A11 (ab175186, 1: 3000), and anti-ACSL4 (ab205199, 1: 1000).

### Dual-luciferase reporter assay

The wild type (wt) and mutant type (mut) of the 3-UTR region of the LINC00616 and TFRC luciferase reporter vectors were designed and synthesized by Guangzhou RiboBio Co., Ltd. (China). The PDLSCs were transfected with miR-370 mimic or nc mimic and wt or mut with LINC00616 or TFRC for 48 h. The PDLSCs were then lysed to detect luciferase activity using a luciferase reporter assay kit (K801-200; BioVision Tech, USA) [17]. Firefly luciferase activity was normalized to Renilla luciferase activity.

### Statistical analysis

All statistical analyses were performed using GraphPad Prism version 8.3 software (GraphPad, USA). Data are presented as mean ± standard deviation from three independent experiments. *P*-values are calculated with Student’s t-test and one-way analysis of variance followed by Tukey’s. A *P*-value less than 0.05 was considered statistically significant.

## Results

### LINC00616 was highly expressed in LPS-PG treated PDLSCs

**Figure 1.**
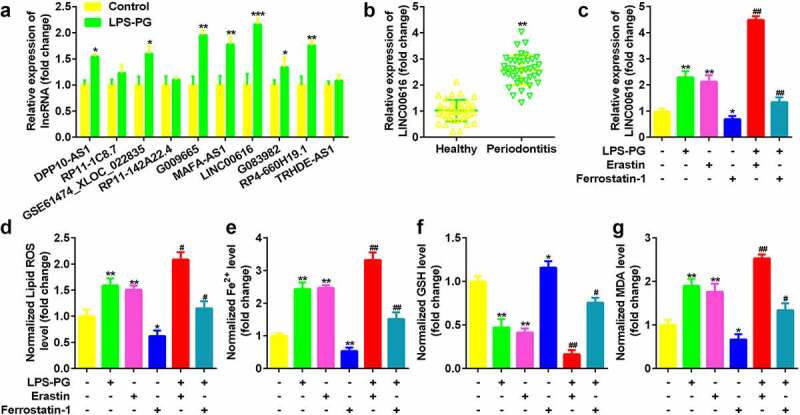
**LINC00616 was highly expressed in LPS-PG-treated PDLSCs with ferroptosis**. (a) Expression of 10 lncRNAs in PDLSCs before and after LPS-PG treatment measured with qRT-PCR. (b) qRT-PCR analyses of LINC00616 expression in patients with periodontitis. (c) qRT-PCR analyses of LINC00616 expression in PDLSCs. (d-g) Levels of ROS, Fe^2+^, GSH, and MDA in PDLSCs.

Expression of 10 lncRNAs which have been identified to be aberrant expressed in periodontitis were measured in LPS-PG treated PDLSCs, among which LIN00616 was markedly upregulated (Figure 1a) [23]. Meanwhile, LINC00616 was also significantly overexpressed in periodontal ligament tissues in patients with periodontitis compared with normal controls (Figure 1b). Moreover, the expression level of LINC00616 was significantly increased by adding the ferroptosis activator erastin, while dramatically decreasing after treatment with the ferroptosis inhibitor Ferrostatin-1 (Figure 1c). The level of GSH in LPS-PG and PDLSCs treated with erastin was significantly decreased and increased, respectively, in the Ferrostatin-1 group; however, ROS, Fe^2+^, and MDA levels were dramatically increased by LPS-PG and erastin and reduced by Ferrostatin-1 (Figure 1d-g).

*P < 0.05, **P < 0.01, ***P < 0.001, compared to the control and healthy groups. ^#^*P* < 0.05, ^##^*P* < 0.01, compared with the LPS-PG group.

### Knockdown of LINC00616 promoted cell viability and inhibited ferroptosis of PDLSCs

**Figure 2.**
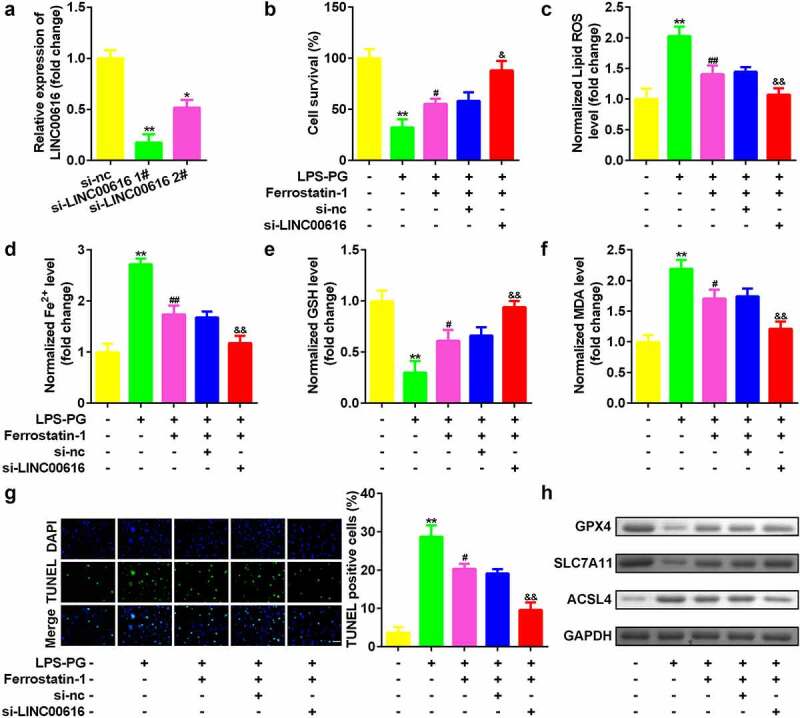
**Knockdown of LINC00616 promoted cell viability and inhibited ferroptosis of PDLSCs**. (a) LINC00616 expression levels were detected by qRT-PCR in PDLSCs after transfection. (b) Cell viability is measured using an MTT assay after transfection in PDLSCs treated with LPS-PG. (c-f) Levels of ROS, Fe^2+^, GSH, and MDA in PDLSCs treated with LPS-PG after transfection. (g) Images and quantized bar chart of TUNEL stained cells. (h) Expression of GPX4, SLC7A11, and ACSL4 proteins detected by western blotting.

Then, we evaluated cellular functions affected by LINC0016 to study the role of LINC00616 in PDLSCs. As shown in Figure 2a, the expression of LINC00616 decreased significantly after transfection of si-LINC00616 into PDLSCs, which was more remarkable in the si-lncLINC00616 1# group. Inhibition of LINC00616 significantly improved cell viability (Figure 2b) and reduced cell death (Figure 2g). Simultaneously, secretion of ROS, Fe^2+^, and MDA was suppressed, while the GSH content increased after LINC00616 was silenced (Figure 2c–f). Furthermore, ferroptosis-related proteins were detected. Treatment with LPS-PG decreased the expression of GPX4 and SLC7A11 and increased the expression of ACSL4, which was dramatically reversed by inhibition of LINC00616 (Figure 2h).

*P < 0.05, **P < 0.01, compared with si-nc and the control group. ^#^*P* < 0.05, ^##^*P* < 0.01, compared with the LPS-PG group. ^&^*P* < 0.05, ^&&^*P* < 0.01, compared with the LPS-PG + Ferrostatin-1 + si-nc group. The scale length is 200 μm.

### LINC00616 sponged miR-370 in PDLSCs

**Figure 3.**
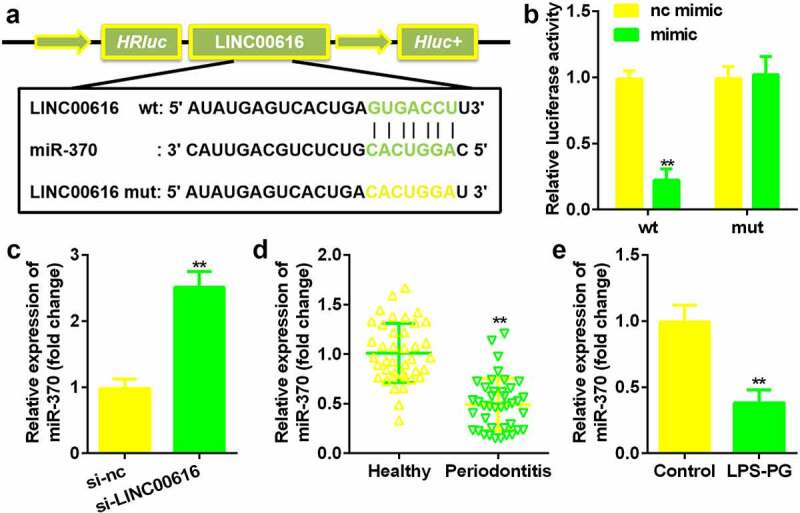
**LINC00616 sponged miR-370 in PDLSCs**. (a) The binding sites between miR-370 and LINC00616 are predicted by StarBase. (b) The dual luciferase reporter assay confirmed that miR-370 is a target of LINC00616 in PDLSCs. (c-e) qRT-PCR analysis for miR-370 expression in periodontal ligament tissues and PDLSCs.

Next, the potential mechanism was investigated. The binding site between LINC00616 and miR-370 was predicted by the StarBase 3.0 online database (Figure 3a). The luciferase activity of the luciferase-labeled miR-370 mimic and the wild-type LINC00616 cotransfection groups was dramatically decreased compared with that of the nc mimic (Figure 3b). Inhibition of LINC00616 upregulated the expression of miR-370 in PDLSCs (Figure 3c). Furthermore, miR-370 expression was markedly suppressed in periodontal ligament tissues from patients with periodontitis and PDLSCs treated with LPS-PG (Figure 3d-e).

**P < 0.01, compared with the nc mimic, si-nc, healthy group, and control group.

### Suppression of miR-370 reversed the effects of LINC00616 on cell viability and ferroptosis in PDLSCs treated with LPS-PG

**Figure 4.**
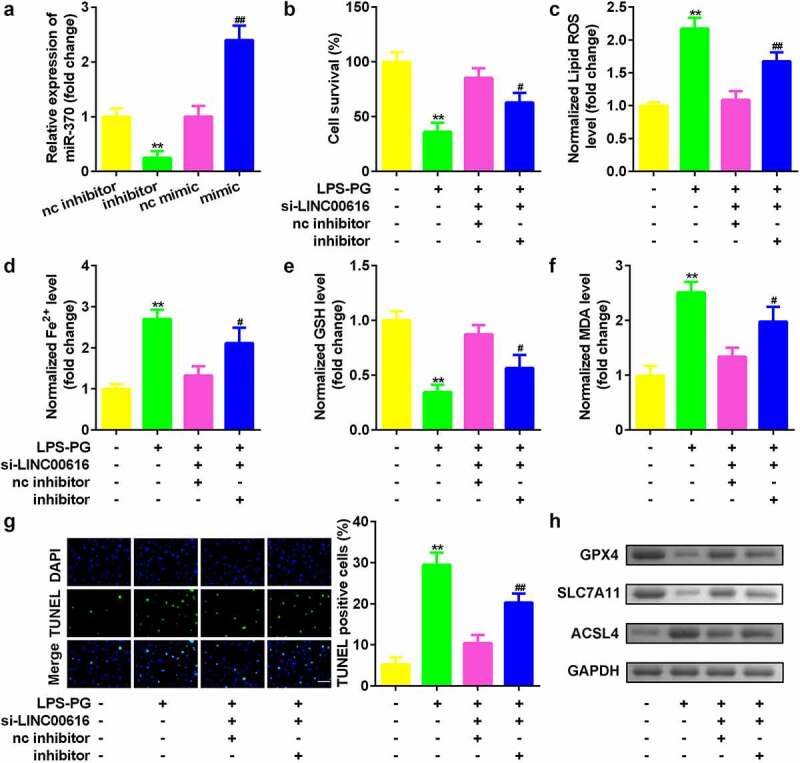
**Suppression of miR-370 reversed the effects of LINC00616 on cell viability, and ferroptosis in PDLSCs treated with LPS-PG**. (a) qRT-PCR analyses of miR-370 expression levels. (b) Cell viability is measured using an MTT assay after cotransfection in LPS-PG treated PDLSCs. (c-f) Levels of ROS, Fe^2+^, GSH, and MDA in PDLSCs treated with LPS-PG after cotransfection. (g) Images and quantized bar chart of TUNEL stained cells. (h) Expression of GPX4, SLC7A11, and ACSL4 proteins detected by western blotting.

Whether miR-370 affect cellular functions was then discussed. The expression of miR-370 was markedly downregulated by the miR-370 inhibitor and upregulated by the miR-370 mimic, suggesting that the PDLSCs were successfully transfected (Figure 4a). Knockdown of miR-370 dramatically alleviated the effects of si-LINC00616 by suppressing cell viability (Figure 4b) and promoting cell death (Figure 4g). Furthermore, downregulation of miR-370 reversed the effects of LINC00616 knockdown on the release of ROS, Fe^2+^, and MDA induced by si-LINC00616 (Figure 4c-f) and the expression levels of GPX4, SLC7A11, and ACSL4 proteins (Figure 4h).

**P < 0.01, compared with nc inhibitor and control group. ^#^*P* < 0.05, ^##^*P* < 0.01, compared with the LPS-PG + si-LINC00616 + nc inhibitor group. The scale length is 200 μm.

### miR-370 directly targeted TFRC in PDLSCs

**Figure 5.**
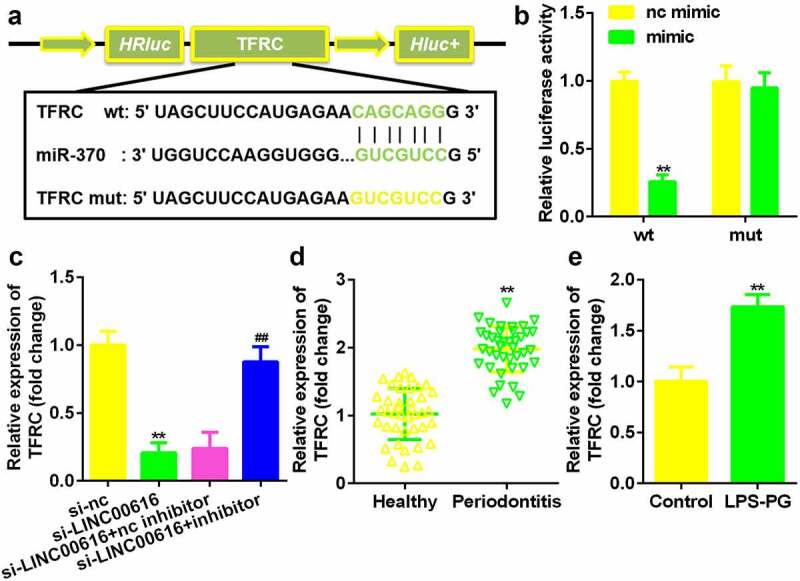
**TFRC was the target gene for miR-370**. (a) Bioinformatics predicted the binding sites between miR-370 and TFRC. (b) A dual-luciferase reporter assay was performed to confirm the association between TFRC and miR-370. (c-e) qRT-PCR analysis for the expression of TFRC in periodontal ligament tissues and PDLSCs.

argetScan 7.2 predicted the binding sites between TFRC and miR-370 (Figure 5a). Luciferase activity was markedly decreased in cells transfected with miR-370 mimic and TFRC 3’-UTR wt (Figure 5b). TFRC expression was markedly reduced by si-LINC00616, which was alleviated by the miR-370 inhibitor (Figure 5c). TFRC was remarkably upregulated in periodontitis ligament tissues of periodontitis patients and LPS-PG treated PDLSCs (Figure 5d-e).

**P < 0.01, compared with nc mimic, si-nc, Healthy, and control group. ^##^*P* < 0.01, compared with the nc mimic group.

### Upregulated TFRC inhibited the effects of downregulated miR-370

**Figure 6.**
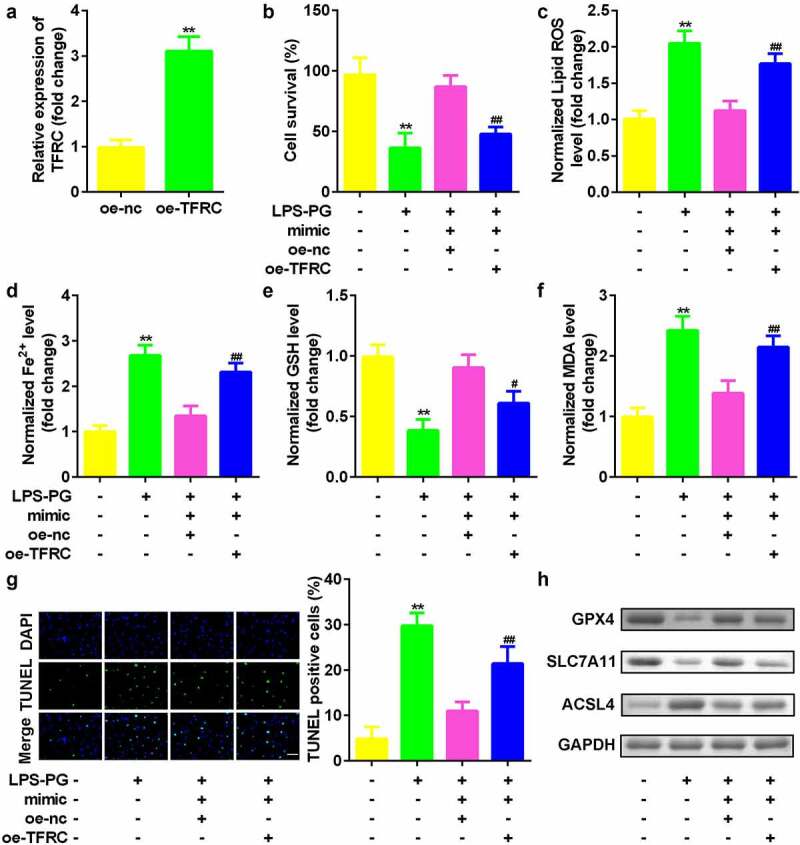
**Upregulated TFRC inhibited the effects of downregulated miR-370**. (a) qRT-PCR analyses of TFRC expression levels. (b) Cell viability is measured using an MTT assay after cotransfection in PDLSCs treated with LPS-PG. (c-f) Levels of ROS, Fe^2+^, GSH, and MDA in PDLSCs treated with LPS-PG after cotransfection. (g) Images and quantized bar chart of TUNEL stained cells. (h) Expression of GPX4, SLC7A11, and ACSL4 proteins detected by western blotting.

Finally, the role of TFRC in regulating PDLSCs was studied. As indicated in Figure 6a, TFRC expression was significantly increased after the oe-TFRC plasmid was transfected into PDLSCs (Figure 6a). Compared with miR-370-overexpressing cells, cotransfection with miR-370 and TFRC vectors significantly inhibited cell viability (Figure 6b) and suppressed ferroptosis (Figure 6c-h).

**P < 0.01, compared with oe-nc and control group. ^#^*P* < 0.05, ^##^*P* < 0.01, compared with the LPS-PG + mimic + oe-nc group. The scale length is 200 μm.

## Discussion

Chronic periodontitis is a common oral chronic inflammatory disease [1,2]. Current periodontitis treatment cannot achieve physiological and functional regeneration of periodontal tissue [3,4]. Therefore, there is an urgent need to identify exact therapeutic targets for periodontitis therapy. Recent evidence suggests that lncRNAs may be associated with periodontitis. For example, the lncRNA AWPPH was overexpressed in plasma from patients with periodontitis compared with healthy controls, which predicts the recurrence of periodontitis [33]. A previous study by Liu et al. suggested that downregulation of the lncRNA MEG3 inhibited the osteogenic differentiation of PDLSCs in periodontitis [17]. Moreover, the lncRNA DCST1-AS1 inhibited the proliferation of PDLSCs in periodontitis [34]. LINC00616 is a newfound lncRNA aberrant expressed in periodontitis; however, the underlying mechanisms remain to be investigated.

Ferroptosis depends on iron and oxidative stress for the regulation of cell death, which differs morphologically, biochemically, and genetically from apoptosis, necrosis, and autophagy [35]. Ferroptosis is a nonapoptotic, nonnecrotic regulator of cell death without rapid depletion of ATP, and inhibitors of apoptosis, autophagy, or necrosis cannot suppress ferroptosis [35]. Overall, ferroptosis leads to cell death through iron-mediated accumulation of lipid ROS, which interferes with cell integrity and membrane fluidity and permeability, and these effects may be the main factors that lead to ferroptosis [8–10]. Furthermore, since iron-dependent oxidative stress and lipid peroxidation are common features of ferroptosis and inflammatory diseases, ferroptosis has been intensively studied in various inflammatory diseases [12]. For example, CD8(+)T cells release interferon-gamma (IFNγ) to inhibit the glutamate-cystine antiporter system xc-, enhance lipid peroxidation, and promote ferroptosis in tumor cells [36]. Ferroptosis also plays a regulatory role in Alzheimer’s disease, Parkinson’s disease, cerebral hemorrhage, and other diseases [12]. Butyrate disrupted iron homeostasis by activating NCOA4-mediated ferritinophagy, leading to ferroptosis in periodontitis [13]. Therefore, we speculated that inhibiting ferroptosis in the course of periodontitis may be a potential therapeutic direction. In the present study, LPS-PG treatment promoted ferroptosis in PDLSCs. Interestingly, knockdown of LINC00616 accelerated cell viability and inhibited ferroptosis of periodontitis model cells, suggesting that inhibition of LINC00616 may alleviate the process of periodontitis.

LncRNAs have been recognized as sponge miRNAs to regulate downstream genes [37]. Bioinformatic analyses indicated that miR-370 was a target miRNA of LINC00616. miR-370 is differentially expressed in cancers and functions as a proto-oncogene or tumor suppressor in diverse tumors [38,39]. However, miR-370 has not been investigated in periodontitis. Our data demonstrated that miR-370 expression was decreased in periodontitis patients and periodontitis model cells. Furthermore, inhibition of miR-370 reversed the effects of silenced LINC00616 on cell viability and ferroptosis. In this study, LINC00616 knockdown may protect PDLSCs by binding to miR-370.

TFRC is a gene that encodes a transferrin receptor and plays an important role in the regulation of iron absorption in the body [40]. Transferrin binds to TFRC on the cell surface and forms a complex to transport iron into cells through endocytosis, increasing the concentration of iron in cells and enhancing the sensitivity of cells to the induction of ferroptosis [40–42]. In this research, TFRC was confirmed to be a target gene of miR-370 and upregulated in periodontitis model cells. TFRC overexpression reversed the effect of miR-370 mimic, suppressed cell viability, and accelerated ferroptosis of PDLSCs. These results suggested that LINC00616 sponged miR-370 to upregulate TFRC and that TFRC may function as a ferroptosis promoter and degrade the cellular functions of PDLSCs, which is consistent with a previous study [32].

## Conclusion

Inhibition of LINC00616 protected PDLSCs by regulating miR-370/TFRC. Knockdown of LINC00616 may be an alternative for the treatment of periodontitis.

## Supplementary Material

Supplemental Material

## References

[1] Abusleme L, Hoare A, Hong BY, et al. Microbial signatures of health, gingivitis, and periodontitis. PERIODONTOL 2000. 2011;50(10). 2021;86 5:57–78. 33690899 10.1111/prd.12362

[2] Hajishengallis G, Lamont RJ. Polymicrobial communities in periodontal disease: their quasi-organismal nature and dialogue with the host. PERIODONTOL 2000. 2021;86 1:210–230. 33690950 10.1111/prd.12371 PMC8957750

[3] Graziani F, Karapetsa D, Alonso B, et al. Nonsurgical and surgical treatment of periodontitis: how many options for one disease? PERIODONTOL 2000. 2017 ;75 1:152–188. 28758300 10.1111/prd.12201

[4] Cobb CM. Lasers and the treatment of periodontitis: the essence and the noise. PERIODONTOL 2000. 2017 ;75 1:205–295. 28758295 10.1111/prd.12137

[5] Song X, Long D. Nrf2 and ferroptosis: a new research direction for neurodegenerative diseases. Front Neurosci. 2020;20(14):267. 10.3389/fnins.2020.00267 PMC7186402 32372896

[6] Yin X, Zeb R, Wei H, et al. Acute exposure of di(2-ethylhexyl) phthalate (DEHP) induces immune signal regulation and ferroptosis in oryzias melastigma. CHEMOSPHERE .2021;265:129053. 10.1016/j.chemosphere.2020.129053 33272674

[7] Ursini F, Maiorino M. Lipid peroxidation and ferroptosis: the role of GSH and GPx4. Free Radic Biol Med. 2020;152:175-185. 10.1016/j.freeradbiomed.2020.02.027 32165281

[8] Xie BS, Wang YQ, Lin Y, et al. Inhibition of ferroptosis attenuates tissue damage and improves long-term outcomes after traumatic brain injury in mice. CNS NEUROSCI THER. 2019;25(4):465–475. 30264934 10.1111/cns.13069 PMC6488926

[9] Mou Y, Wang J, Wu J, et al. Ferroptosis, a new form of cell death: opportunities and challenges in cancer. J HEMATOL ONCOL. 2019;12(1):34. 30925886 10.1186/s13045-019-0720-y PMC6441206

[10] Liang C, Zhang X, Yang M, et al. Recent progress in ferroptosis inducers for cancer therapy. ADV MATER. [Journal Article; Review]. 2019; 01;31 51:e1904197. 31595562 10.1002/adma.201904197

[11] Weiland A, Wang Y, Wu W, et al. Ferroptosis and its role in diverse brain diseases. MOL NEUROBIOL. 2019;56(7):4880–4893. 30406908 10.1007/s12035-018-1403-3 PMC6506411

[12] Mao H, Zhao Y, Li H, et al. Ferroptosis as an emerging target in inflammatory diseases. Prog Biophys Mol Biol. 2020;1(155):20-28. 10.1016/j.pbiomolbio.2020.04.001 32311424

[13] Zhao Y, Li J, Guo W, et al. Periodontitis-level butyrate-induced ferroptosis in periodontal ligament fibroblasts by activation of ferritinophagy. Cell Death Discov. 2020;6 1:119. 33298848 10.1038/s41420-020-00356-1 PMC7655826

[14] Bullon P, Cordero MD, Quiles JL, et al. Mitochondrial dysfunction promoted by porphyromonas gingivalis lipopolysaccharide as a possible link between cardiovascular disease and periodontitis. Free Radic Biol Med. 2011:1336–1343. 21354301 10.1016/j.freeradbiomed.2011.02.018

[15] Sayad A, Ghafouri-Fard S, Shams B, et al. Blood and tissue levels of lncRNAs in periodontitis. J CELL PHYSIOL. 2020;235 12:9568–9576. 32372456 10.1002/jcp.29764

[16] Zou Y, Li C, Shu F, et al. lncRNA expression signatures in periodontitis revealed by microarray: the potential role of lncRNAs in periodontitis pathogenesis. J CELL BIOCHEM. 2015;116(4):640–647. 25399840 10.1002/jcb.25015

[17] Liu Y, Liu C, Zhang A, et al. Down-regulation of long non-coding RNA MEG3 suppresses osteogenic differentiation of periodontal ligament stem cells (PDLSCs) through miR-27a-3p/IGF1 axis in periodontitis. Aging (Albany NY). 2019;11(15):5334–5350. 31398715 10.18632/aging.102105 PMC6710065

[18] Zhu Y, Ai R, Ding Z, et al. LncRNA-01126 inhibits the migration of human periodontal ligament cells through MEK/ERK signaling pathway. J PERIODONTAL RES. 2020;55(5):631–641. 32240552 10.1111/jre.12749

[19] Dexheimer PJ, MicroRNAs: CL. From mechanism to organism. Front Cell Dev Biol. 2020;8 409. 32582699 10.3389/fcell.2020.00409 PMC7283388

[20] Jin SH, Zhou JG, Guan XY, et al. Development of an miRNA-array-based diagnostic signature for periodontitis. FRONT GENET. 2020;11:577585. 10.3389/fgene.2020.577585 PMC7772397 33391341

[21] Tay Y, Rinn J, Pandolfi PP. The multilayered complexity of ceRNA crosstalk and competition. NATURE. 2014;505 7483:344–352. 24429633 10.1038/nature12986 PMC4113481

[22] Qi X, Zhang DH, Wu N, et al. ceRNA in cancer: possible functions and clinical implications. J MED GENET. 2015;52 10:710–718. 26358722 10.1136/jmedgenet-2015-103334

[23] Liu Y, Liu Q, Li Z, et al. Long non-coding RNA and mRNA expression profiles in peri-implantitis vs periodontitis. J PERIODONTAL RES. 2020;55(3):342–353. 31853997 10.1111/jre.12718

[24] Lee JS, Lee JB, Cha JK, et al. Chemokine in inflamed periodontal tissues activates healthy periodontal-ligament stem cell migration. J CLIN PERIODONTOL. 2017;44(5):530–539. 28207939 10.1111/jcpe.12710

[25] Tang J, Wu T, Xiong J, et al. Porphyromonas gingivalis lipopolysaccharides regulate functions of bone marrow mesenchymal stem cells. Cell Prolif. 2015;48(2):239–248. 25676907 10.1111/cpr.12173 PMC6496502

[26] Xing H, Taguchi Y, Komasa S, et al. Effect of porphyromonas gingivalis lipopolysaccharide on bone marrow mesenchymal stem cell osteogenesis on a titanium nanosurface. J PERIODONTOL. 2015;86(3):448–455. 25494660 10.1902/jop.2014.140386

[27] Chen Q, Liu X, Wang D, et al. Periodontal inflammation-triggered by periodontal ligament stem cell pyroptosis exacerbates periodontitis. Front Cell Dev Biol.[Journal Article]. 2021 2021 January 20;9:663037. 33869229 10.3389/fcell.2021.663037 PMC8049442

[28] Du W, Wang L, Liao Z, et al. Circ_0085289 alleviates the progression of periodontitis by regulating let-7f-5p/SOCS6 pathway. INFLAMMATION. 2021;44 4:1607–1619. 33710445 10.1007/s10753-021-01445-8

[29] Ye J, Coulouris G, Zaretskaya I, et al. Primer-BLAST: a tool to design target-specific primers for polymerase chain reaction. BMC BIOINFORMATICS. 2012;13 1:134. 22708584 10.1186/1471-2105-13-134 PMC3412702

[30] Wang C, Wang J, Shen X, et al. LncRNA SPOCD1-AS from ovarian cancer extracellular vesicles remodels mesothelial cells to promote peritoneal metastasis via interacting with G3BP1. J Exp Clin Cancer Res. 2021;40(1):101. 33726799 10.1186/s13046-021-01899-6 PMC7968157

[31] He R, Cui M, Lin H, et al. Melatonin resists oxidative stress-induced apoptosis in nucleus pulposus cells. LIFE. 2018;199:122–130. 10.1016/j.lfs.2018.03.020 29526797

[32] Liu Y, Yang H, Wen Y, et al. Nrf2 inhibits periodontal ligament stem cell apoptosis under excessive oxidative stress. INT J MOL SCI. 2017;18(5):1076. 10.3390/ijms18051076 PMC5454985 28513573

[33] Wang X, Ma F, LncRNA JP. AWPPH overexpression predicts the recurrence of periodontitis. Biosci Rep. 2019;39(7):10.1042/BSR20190636. PMC6658719 31289125

[34] Wang X, LncRNA WY. DCST1-AS1 inhibits PDLCs’ proliferation in periodontitis and may bind with miR-21 precursor to upregulate PLAP-1. J PERIODONTAL RES. 2021;56 2:256–264. 33533513 10.1111/jre.12809

[35] Su LJ, Zhang JH, Gomez H, et al. Reactive oxygen species-induced lipid peroxidation in apoptosis, autophagy, and ferroptosis. OXID MED CELL LONGEV. 2019;2019:5080843. 31737171 10.1155/2019/5080843 PMC6815535

[36] Wang W, Green M, Choi JE, et al. CD8(+) T cells regulate tumour ferroptosis during cancer immunotherapy. NATURE. 2019;569(7755):270–274. 31043744 10.1038/s41586-019-1170-y PMC6533917

[37] Wan J, Liu B. Construction of lncRNA-related ceRNA regulatory network in diabetic subdermal endothelial cells. BIOENGINEERED. 2021;12 1:2592–2602. 34124997 10.1080/21655979.2021.1936892 PMC8806614

[38] Chen J, Liu G, Wu Y, et al. CircMYO10 promotes osteosarcoma progression by regulating miR-370-3p/RUVBL1 axis to enhance the transcriptional activity of beta-catenin/LEF1 complex via effects on chromatin remodeling. MOL CANCER. 2019;18(1):150. 31665067 10.1186/s12943-019-1076-1 PMC6819556

[39] Wei CY, Zhu MX, Lu NH, et al. Circular RNA circ_0020710 drives tumor progression and immune evasion by regulating the miR-370-3p/CXCL12 axis in melanoma. MOL CANCER. 2020;19(1):84. 32381016 10.1186/s12943-020-01191-9 PMC7204052

[40] Wu J, Minikes AM, Gao M, et al. Intercellular interaction dictates cancer cell ferroptosis via NF2-YAP signalling. NATURE. 2019;572(7769):402–406. 31341276 10.1038/s41586-019-1426-6 PMC6697195

[41] Jabara HH, Boyden SE, Chou J, et al. A missense mutation in TFRC, encoding transferrin receptor 1, causes combined immunodeficiency. NAT GENET. 2016;48(1):74–78. 26642240 10.1038/ng.3465 PMC4696875

[42] Guo S, Chen Y, Xue X, et al. TRIB2 desensitizes ferroptosis via betaTrCP-mediated TFRC ubiquitiantion in liver cancer cells. Cell Death Discov. 2021;7(1):196. 34315867 10.1038/s41420-021-00574-1 PMC8316344

