# Supplementary figures and images for: Long non-coding RNA LINC00616 promotes ferroptosis of periodontal ligament stem cells via the microRNA-370 / transferrin receptor axis
Source: Bioengineered. 2022 May 25;13(5):13070–81. doi: 10.1080/21655979.2022.2076508 (PMC9276003; doi:10.1080/21655979.2022.2076508)

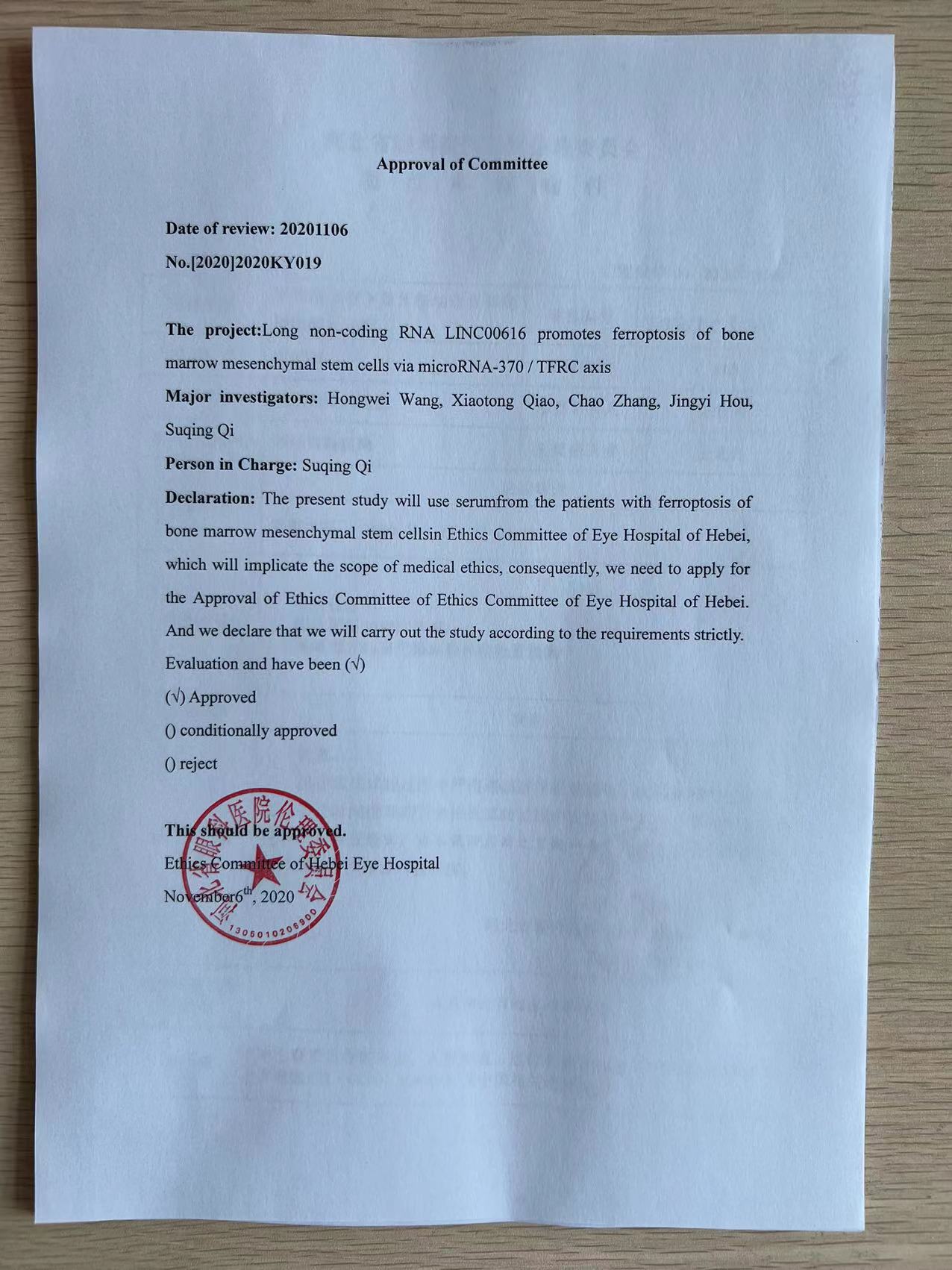

Supplement: Supplemental Material [file KBIE_A_2076508_SM1673.zip › ethical approvement.jpg]
